# Using Convolutional Neural Network and a Single Heartbeat for ECG Biometric Recognition

**DOI:** 10.3390/e23060733

**Published:** 2021-06-09

**Authors:** Dalal A. AlDuwaile, Md Saiful Islam

**Affiliations:** Computer Science Department, College of Computer and Information Sciences, King Saud University, Riyadh 11543, Saudi Arabia; saislam@ksu.edu.sa

**Keywords:** biometrics, deep learning, convolutional neural network, ECG signal, continuous wavelet transformation

## Abstract

The electrocardiogram (ECG) signal has become a popular biometric modality due to characteristics that make it suitable for developing reliable authentication systems. However, the long segment of signal required for recognition is still one of the limitations of existing ECG biometric recognition methods and affects its acceptability as a biometric modality. This paper investigates how a short segment of an ECG signal can be effectively used for biometric recognition, using deep-learning techniques. A small convolutional neural network (CNN) is designed to achieve better generalization capability by entropy enhancement of a short segment of a heartbeat signal. Additionally, it investigates how various blind and feature-dependent segments with different lengths affect the performance of the recognition system. Experiments were carried out on two databases for performance evaluation that included single and multisession records. In addition, a comparison was made between the performance of the proposed classifier and four well-known CNN models: GoogLeNet, ResNet, MobileNet and EfficientNet. Using a time–frequency domain representation of a short segment of an ECG signal around the R-peak, the proposed model achieved an accuracy of 99.90% for PTB, 98.20% for the ECG-ID mixed-session, and 94.18% for ECG-ID multisession datasets. Using the preprinted ResNet, we obtained 97.28% accuracy for 0.5-second segments around the R-peaks for ECG-ID multisession datasets, outperforming existing methods. It was found that the time–frequency domain representation of a short segment of an ECG signal can be feasible for biometric recognition by achieving better accuracy and acceptability of this modality.

## 1. Introduction

The recent explosive evolution in science and technology has raised security standards, rendered classical security methods, such as keys, passwords, PIN codes, and ID cards, unsatisfactory and opened the door for new technologies. Biometric authentication is one approach that provides a unique method for identity recognition. This approach uses metrics related to human characteristics, such as facial features [1,2], fingerprints [3,4], hand-geometry [5], handwriting [6,7], the iris [8,9], speech [10,11], and gait [12,13] for identification and verification. However, these traditional biometric modalities have proved to be vulnerable, as they can be easily replicated and used fraudulently [14]. For instance, the face is vulnerable to artificial masks, fingerprints and hand features can be recreated by latex, handwriting and voice are easy to mimic, and the iris can be faked by using contact lenses with copied iris features printed on it.

In recent times, physiological signals, such as electroencephalogram (EEG) signals produced by the brain [15,16] and electrocardiogram (ECG) signals produced by the heart [17,18,19] have become popular for biometric recognition. As biometric modalities, their main advantages are that the brain and heart are confined inside the body’s structure, making them secure against any tempering and difficult to simulate or copy. Furthermore, they have liveness properties, as these signals can be captured from living individuals only. Among these physiological signals, good quality ECG signals can be easily captured from fingers [20,21], unlike EEG signals, which are hard to capture without sophisticated equipment. Hence, the ECG signal could be more acceptable as a biometric modality for commercial and public applications. ECG signals have been studied and presented as a biometrics modality for the last two decades. They have been proven to have the needed properties for a reliable identification process, such as universality, performance, uniqueness, robustness to attacks, liveness detection, continuous authentication, and data minimization [22,23].

Recently, deep-learning techniques have effectively used ECG signals for improving recognition performance [19,21]. However, one of the factors that affect the accuracy of biometric recognition is the length of the ECG signal used. Generally, most of the existing works use a long segment of a signal consisting of several heartbeats; it requires a long time to capture and process [24,25], which may not be acceptable by users in commercial applications. A limited number of works studied the effect of the length of the segment on the recognition process. Moreover, ECG signals of a person can also vary due to different physiological and mental conditions, which become more apparent in signals captured in different sessions [26]. Accordingly, multisession analysis reveals the effectiveness and robustness of any ECG-based recognition system. Based on the results reported to date, most of the existing, deep learning-based methods usually consider only single-session records, and a limited number of studies consider multisession analysis [27,28].

This paper investigates how a time–frequency domain representation of a short segment of an ECG signal can be effectively used for biometric recognition, using deep learning techniques to improve the acceptability of this modality. Capturing the right part of the signal can improve the quality of the segments, which improves the learning process as well and increases the classification accuracy without needing a large number of heartbeat samples. The speed of the classification also increases, and the identification system becomes more reliable and acceptable. Based on the investigation, a small convolutional neural network (CNN) was designed for biometric recognition, using short segments of a heartbeat signal around the R-peak. The segment was presented as input images after applying continuous wavelet transformation (CWT). Four pre-trained networks of different models (GoogLeNet, ResNet, MobileNet and EfficientNet) were also used. The effect of the segmentation methods using single session analysis was investigated, using the Physikalisch Technische Bundesanstalt (PTB) [29] dataset, an ECG dataset that is widely used for deep convolutional recognition models. The ECG-ID [30] dataset was used for the multisession analysis. The main contributions of this paper can be identified as follows:

1. We investigate the effectiveness of time–frequency domain representation of a short segment of an ECG signal (0.5-second window around the R-peak) for improved biometric recognition. The significance of this finding is that it improves the acceptability of an ECG signal as a biometric modality, which can be used for a liveness test.
2. A small convolutional network (CNN) is designed to learn less complex decision boundaries in the transferred domain to achieve better generalization capability and at the same time to avoid overfitting.
3. This study investigates the effects of different types of segments of ECG signal, such as fixed-length, variable-length, blind, and feature-dependent segments, on the deep learning-based ECG recognition process.
4. The effectiveness of the short segment is also investigated, using a multisession database to ensure its viability in biometric recognition over time. The viability of a short segment can help to develop a robust, reliable, and acceptable authentication system. It can also make this modality practical to fuse with other modalities, especially the fingerprint, to improve the robustness and security of biometrics in general [25,31].

The rest of the paper is organized as follows. Section 2 presents recent state-of-the-art approaches for ECG biometric recognition that apply deep learning, using different segments and lengths of the signal. In Section 3, we describe the deep learning-based biometric recognition method. In Section 4, we describe the experimental protocol for testing the effectiveness of the proposed method. Experimental results and discussion are given in Section 5. Finally, the conclusion and future works are given in Section 6.

## 2. Related Works

This section presents recent works on ECG biometric systems that used deep learning approaches for biometric recognition. We specifically reviewed the performance of the state-of-the-art methods for using different types of segments of ECG signals (e.g., blind segment or fiducial point-based segment) and the length of the segment. We also reviewed whether testing was carried out using the same session or multisession records.

Feature engineering-based Multilayer Perceptrons (MLP) were previously used in ECG signal classification [32]. SVM was used as a classifier in [33] that obtained 93.15% accuracy for 50 subjects from the PTB dataset and another 140 subjects from a private dataset. SVM was also used to classify 10 subjects from the PTB dataset in [34], obtaining 97.45% accuracy. Recently, deep learning-based methods have become popular for ECG biometrics recognition [19,21]. An inception network was used as a classifier in [32] with an accuracy of 97.84%. In [35], RNN was used as a feature extractor, along with GRU and LSTM as classifiers, and was tested with 90 subjects from the ECG-ID database and 47 subjects from the MIT-BIH database to obtain approximately 98% accuracy. The model proposed in [36] used a multiresolution, 1D CNN with an overall accuracy of 93.5% on eight ECG datasets with subjects ranging from 18 to 47. CNN was also used as a classifier in [37] and obtained almost 97% accuracy for identifying 90 subjects from the ECG-ID dataset. A fusion technique between 1D and 2D CNNs was presented in [38] to verify 46 subjects from UofTDB, obtaining a 13% equal error rate (EER), and for verifying 65 subjects from the CYBHi dataset to obtain a 1.3% equal error rate (EER). Multiple single session datasets were used to test the cascade CNN proposed in [39], where the number of subjects did not exceed 28. This model resulted in accuracy ranges from almost 92% to 99.9%, depending on the datasets that were tested. A private dataset was collected to test the model proposed in [27], for which a CNN was used to classify 800 subjects with 2% EER. In [40], a 98.84% classification accuracy was obtained, using CNN on 52 subjects from the PTB dataset and 99.2% on 18 subjects from the MIT-BIH dataset.

As mentioned previously, there is no proper investigation on the impact of segment length on the recognition process in the literature. The segmentation was mentioned only as part of the method been used. Several studies used fixed-length, blind segmentation as in [41], for which segments of 15 seconds were used. A large, 10 s window captured blindly was used in [42] and [24]. A relatively small blind segmentation of 2 s and 3 s of ECG data was used in [36] and [37], respectively. R-peak dependent segmentation was widely used. Segment durations of 0.2 s and 1 s around the R-peaks were used in [43]. A 0.65 s segment around the R-peak was used in [44], and 3.5 s segments around R-peaks were used in [45]. Segments of lengths 0.5, 1.6, and 2 s for three different datasets were tested in [46]. In [47], a large 11 s segment around the R-peak was used. The model in [38] used 3.5 s segments around the R-peak from the UofTDB database and 0.8 s segments around the R-peak from the CYBHi database. Segments of 3 s in length captured around the R-peak were used in [27]. A fixed-size segmentation window around the R-peak was also used in [40], and the size of the window was calculated using the average of the first 5 RR intervals in [48]. The RR-interval was used as the segment in [33,34] and [40].

Most of the available deep learning base methods used single session datasets for the evaluation of biometric recognition performance, as shown in Table 1. In [33,37,38,49,50], cross-session and same session records were tested. Table 1 summarizes state-of-the-art approaches for the ECG biometrics based on segmentation type and length, feature extractor, and classification scheme.

## 3. Method

**Figure 1:**
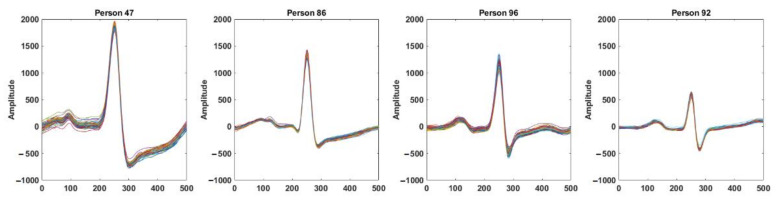
Segments of ECG signal around the R-peaks for four different individuals in the same ECG record. Each subfigure shows the intra-individual similarities; inter-individual differences among the individuals can be observed from four subfigures.

**Figure 2:**
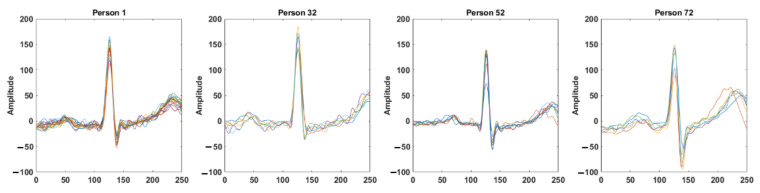
Segments of ECG signal around the R-peaks for four different individuals in different ECG records obtained in different sessions. Each subfigure shows the intra-individual similarities; inter-individual differences among the individuals can be observed from four subfigures.

ECG is a continuous and semi-periodic signal representing the electrical activities of the heart. For a healthy individual, a single heartbeat contains all of the morphological features. Each heartbeat signal consists of a sequence of P, QRS, and T waves occurring repeatedly. The duration of heartbeats of an individual varies due to different physiological and mental conditions. However, for biometric recognition, the invariant segment of the signal is required. It is observed that for a healthy person, the signal around the QRS complex is the most invariant segment, which preserves its shape over time and is the most distinctive from other individuals. Figure 1 shows the intra-individual similarities and inter-individual differences of the segments obtained from four different individuals. Here, each of the subfigures show 0.5-second windows of the signal around the R-peaks of an ECG record obtained from the PTB dataset [29]. Although the morphology of the signal for a particular person remains invariant, the inter-individual difference is noticeable. In Figure 2, we plotted 0.5 s windows of signals around the R-peaks obtained from different ECG records on a particular individual from different sessions in the ECG-ID database [30]. Only one segment is taken from each record. The intra-individual correlations of the signal among different sessions and the inter-individual differences can be observed.

**Figure 3:**
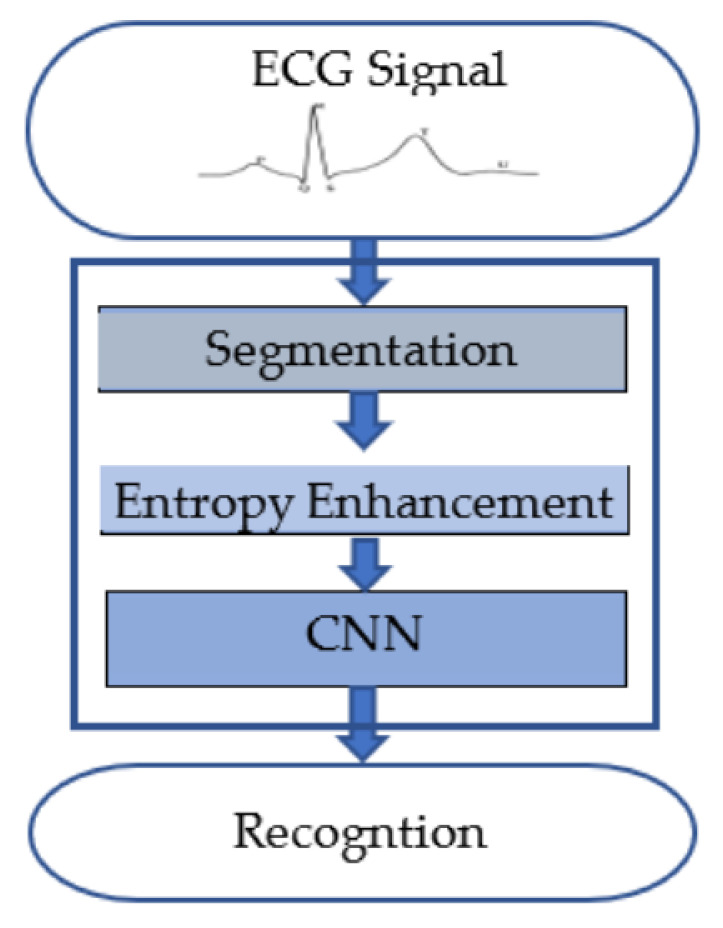
Block diagram of the proposed deep learning-based biometric recognition process.

The recognition process uses a small segment of the ECG signal as the input, which is then transformed into Continuous Wavelet Transformation (CWT) images and used by the convolutional neural network (CNN) for the recognition of the individual. Figure 3 shows the block diagram for biometric recognition. We obtain a segment of the ECG signal as the preprocessing step as discussed in Section 3.1. Section 3.2 describes the Continuous Wavelet Transformation method for the time–frequency domain representation of a segment of an ECG signal. Finally, the CNN-based deep learning method is discussed in Section 3.3.

### 3.1. Segmentation of an ECG Signal

**Figure 4:**
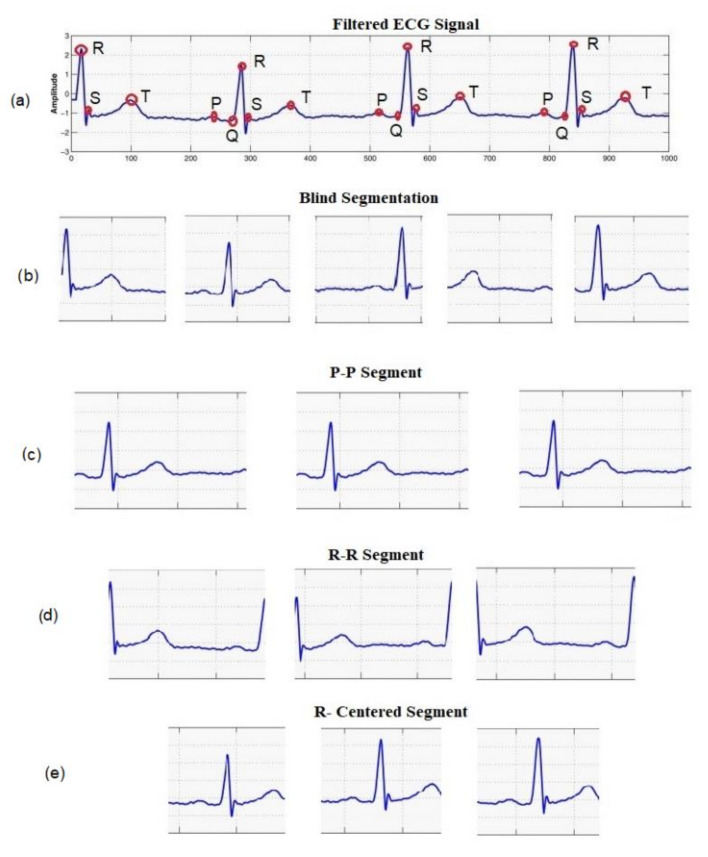
Examples of segmentation methods: Blind and HB segmentation. (**a**) Original signal with P, Q, R, S, and T peaks, (**b**) blind segments, (**c**) PP-interval segments, (**d**) RR-interval segments, and (**e**) R-centered segments.

There are different ways to obtain a segment of the ECG signal for biometric recognition, as shown in Figure 4. A segment can be captured for a constant period, obtaining an arbitrary fragment of a segment (blind segment), which may exclude certain heartbeat features or may include redundant features. Another way is to depend on fiducial points that capture the essential features of a heartbeat. Hence, three different types of segments are considered: (i) R-centered segments, which are taken in a window around the R-peak, (ii) R-R segments, and (iii) P-P segments.

The P and R segments were detected following the method presented in [53,54]. In this method, the approximate location of R-peak is detected as the local minima of the signal’s curvatures, using a sliding window and an adaptive threshold. Then a search back in the preprocessed signal within a window of the samples around the approximated locations is applied to obtain the final location of the R-peak. The P-peak is identified to the left of the R-peak as the maximum within a window of 245 ms in the signal obtained by the augmented-Hilbert transform [54].

### 3.2. Entropy Enhancement

Although a short segment of the ECG signal could be helpful for the acceptability of this biometric modality, the information content of such a segment is rather limited due to the finite time–domain representation. The information content offered by the signal can be analyzed by biometric system entropy (BSE) [55,56]. Here, BSE measures the entropy by using Kullback–Leibler (KL) divergence of the signal (*s*) from two different distributions of genuine user scores (*f_G_*) and imposter scores (*f_I_*) obtained from a given set of ECG samples as defined below:

(1) $$\mathrm{BSE}\left(s\right)=\int f_{G}log\left(\frac{f_{G}}{f_{I}}\right)dG$$

Frequency analysis of the signal allows us to enhance entropy by representing the signal in the infinite frequency domain. Although various methods exist [25,57], continuous wavelet transformation (CWT) proposes a method of representation of the signal *s*(*t*) in a 2D time–frequency domain, using mother wavelet functions + *φ*:

(2) $$s_{cwt}\left(a,b\right)=\frac{1}{\sqrt{a}}\int s\left(t\right)\varphi \left(\frac{t-b}{a}\right)dt$$

where *a* is the scale factor and *b* is the shift of the wavelet function.

**Figure 5:**
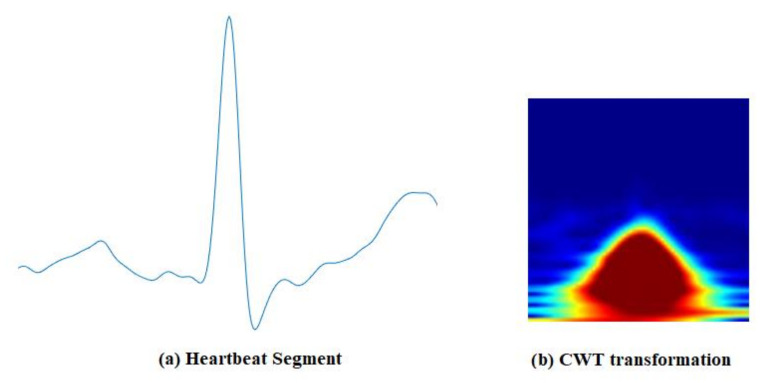
Continuous Wavelet Transformation: (**a**) A segment of ECG signal, (**b**) CWT image.

**Figure 6:**
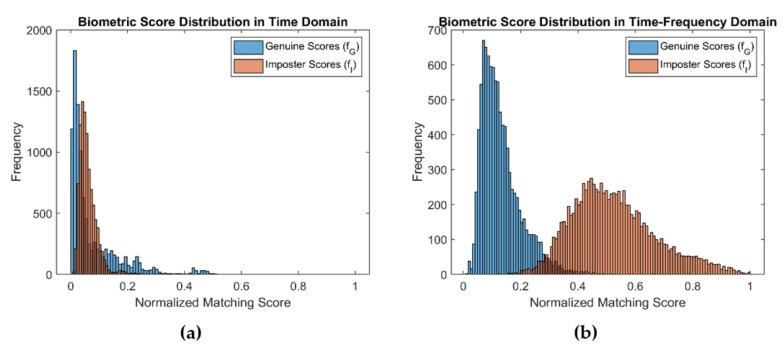
Distribution of genuine score and imposter scores of ECG signals (**a**) in time–domain and (**b**) CWT representation.

The time–frequency representation of the signal is obtained by decomposing it at different time scales, each of which represents a specific frequency range in the time–frequency plane. This representation, which consists of the absolute value of the CWT coefficients of the signal, is called a scalogram [58]. Figure 5 shows time–frequency representations of segmented ECG signals, using CWT. To examin the BSE of the time–domain signal and its CWT representation, we created two distributions of 9900 biometric scores for each from ECG signals obtained from the PTB [29] dataset. Figure 6a,b shows the distributions of biometric scores for genuine users and imposters in time and time–frequency domains, respectively. The entropy computed by using Equation (1) was 1.44 and 3.15 for the time and time–frequency domains, respectively, indicating significant enhancement of BSE.

### 3.3. Deep Learning

Different deep learning-based methods, especially CNN models, are becoming popular in ECG-based biometric recognition [24,39]. To achieve optimal classification accuracy, the size of the training datasets used for CNN is consistently increasing, which, in turn, has caused the volume of CNN models proposed for image classification to continuously grow larger and require more learning time and space. By enhancing the entropy, the CWT representation makes the decision boundary much simpler as shown in Figure 6. Hence, a simpler CNN could effectively learn the decision boundary with more generalization capability. On the other hand, very deep CNN models, designed for complex image learning, could suffer from overfitting due to the fact that the CWT representation transfers the signal into less complex images, yielding decision boundaries with a smaller degree of nonlinearity.

**Figure 7:**
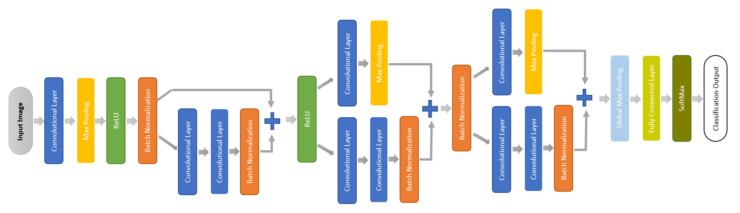
Block digram of the proposed small CNN.

We designed a small CNN that depends on the quality of short ECG segments with enhanced entropy to learn the relatively simpler decision boundary offered by CWT representation. The proposed small CNN is a network with fewer layers as presented in Figure 7. It starts with a convolutional layer with a small filter size followed by max-pooling layers, a rectified linear unit (ReLU), and batch normalization. Then a residual block uses the max-pooling layer as a skip connection when the gradient becomes very small and prevents the weights from changing in value. After that comes a fully connected layer followed by SoftMax and classification layers, which predict each test sample label and determine the classification accuracy. Table 2 presents the detailed parameters of the network.

In addition to the small CNN network, a deep-learning method known as transfer learning, where a previously trained model works as a starting point for a model to be used in a new task [59], was used. Transfer learning has a lower computational cost than training a new model from scratch. GoogLeNet [60], ResNet [61], EfficientNet [62] and MobileNet [63] architectures were chosen as the pre-trained models in this study. The main idea of GoogLeNet is the inception layers. There are nine inception layers; each layer has parallel convolutional layers with different filter sizes to simultaneously maintain the resolution for small information and cover a larger area in the image. The residual block is a solution for when the gradient becomes very small and prevents weights from changing in value. MobileNet depends on depthwise separable convolution to reduce the model size and complexity, which makes it useful for mobile and embedded vision applications. EfficientNet uses compound coefficient to scale up width, depth or resolution uniformly. All networks were originally trained to classify images into one of 1000 categories. In this study, the pre-trained network’s output layer was replaced with the number of classes that needed to be predicted for each experimental scenario. Table 3 presents the number of layers, learnable parameters, and average training time for each model.

## 4. Experiments

### 4.1. Datasets

We used two datasets in this study: (i) the PTB dataset and (ii) the ECG-ID dataset. These two databases are popular PhysioNet databases that several researchers used to test the performance of ECG-based authentication and identification algorithms. These datasets include data with different sampling rates, leads, resolutions, and lengths. Additionally, they contain both normal and abnormal signals, which aids in testing the generalized performance of the networks.

The PTB is a public database with diverse profile information, such as gender, age, health information, and different ECG lengths obtained from 290 subjects sampled at 1 kHz. Subjects 124, 132, 134, and 161 are not available in the database. Each record includes 12 lead ECG signals. We only used a single lead (lead i) as the raw signal source. In this study, we considered 100 subjects.

The ECG-ID database contains ECG recordings obtained from 90 healthy individuals. Each recording lasts for 20 seconds and is digitized at 500 Hz. Some subjects’ records were collected in a one-day session, while others were collected in multiple periodic sessions over six months.

### 4.2. Equipment

The experiment was conducted using MATLAB and a PC with the following features:

- Intel^®^ Core i5-8600K CPU @ 3.60 GHz 6-core machine;
- 240 GB of DDR4 RAM;
- One GTX 1080 Ti GAMING OC 11 GB.

### 4.3. Experimental Protocol

**Figure 8:**
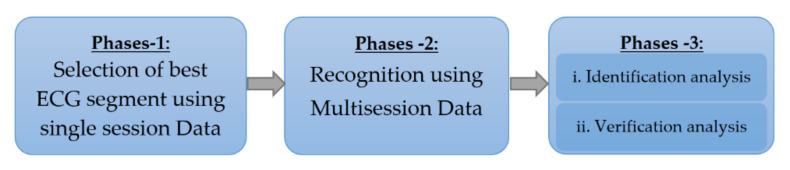
Block diagram of experimental protocol.

The experiments were conducted in three phases to investigate the effect of different ECG segments on the recognition process, as illustrated in Figure 8. The first phase examined how different segmentation types with different lengths affected the recognition accuracy, using the PTB dataset. In the second phase, the best segmentation and length option was used to examine the classification performances of the networks, using the multisession ECG-ID dataset. In the third phase, the classification results were analyzed to determine the identification and verification performance by scenarios as suggested in [15,64].

Phase-1: Segmentation and length analysis

Records of ECG signals are segmented using different methods:

- Blind segmentation: A preprocessed signal is blindly divided into segments of equal durations. To examine the effect of different segment sizes, we performed segmentation with different window sizes, such as 0.5, 1, 1.5, 2, 2.5, and 3 s.
- Heartbeat segmentation: An ECG record is divided into segments based on different fiducial points, such as the P- and R-peaks in the QRS complex, producing the (i) R-centered segment, (ii) R-R segment, and (iii) P-P segment. We divided the signal using three different window sizes, 0.5, 0.75, and 1 s, around each R-peak in the R-centered segment. To balance the samples, only subjects in which the P-peak was detectable were considered in this phase. We selected 100 records with a detectable P-peak.

Phase-2: Recognition using multisession data

The segment of the ECG signal with the highest recognition accuracy from phase one was used to examine the performance of the recognition process in multisession scenarios using the ECG-ID database. Since this dataset contains 90 subjects, we used 90 class classification problem, using the following three scenarios:

- Single session: To support the findings of phase-1 and to compare with other methods (using single-session data only), we used one record of each subject from ECG-ID in this scenario and divided them into training and test sets.
- Mixed session: We collected segments from ECG signals in different sessions and mixed them together before dividing them into training and test sets.
- Multisession: The training and test segments were collected from ECG signals in different sessions without mixing them. In the ECG-ID dataset, all subjects have at least two records, except subject 74. For subject 74, we used the same record for both sessions, but the segments were randomly divided into training and test segments. This resulted in 90 classifiable subjects.

Phase-3: Identification and verification analysis

Biometric recognition systems are generally used in two different modes in practical applications: (i) identification (one to many matching), (ii) verification (one to one matching). We used the results obtained by the biometric recognition process to analyze the identification and verification performance by scenarios as suggested in [15,64]. In this process, we used the results of classification using the multisession data only. The identification analysis aims to identify each of the 90 subjects correctly. On the other hand, the verification analysis aims to identify one subject versus all other 90 subjects. In this case, there were only two classes: genuine (target subject) and imposter (all other subjects).

### 4.4. Network Training and Testing

The segmentation process was followed by data augmentation to increase the number of segments and balance the number of samples among the subjects. New segments were generated as needed from the original segments, where the new segment was the average of 10 randomly selected segments from the same individual. We used 100 heartbeat segments for each individual, leading to a total number of 10,000 segments in phase one and 9000 segments in total for the single-session, mixed-session, and multisession analyses.

A 10-fold cross-validation method was used to reduce the training set’s generalization error. The cross-validation test generates ten different results, the average of which is the final accuracy of the classification task. The 10-fold cross-validation was used in the phase one analysis and the first two session analysis scenarios: single and mixed sessions.

For the multisession analysis, a 2-fold cross-validation was conducted, where the first iteration of training used segments from one session, while the testing set used segments from other sessions. Sessions were then exchanged for the second iteration, which generated two different results. The average of the two outcomes was the final accuracy.

For training the CNNs, stochastic gradient descent with minimum batches of size 150 and a momentum coefficient of 0.9 were used. A learning rate of 0.001 was considered during 80 training epochs. We tried higher values for training epochs, but no improvements were noticed.

### 4.5. Evaluation

We used the classification results for a network to compute the true positive (TP), true negative (TN), false positive (FP), and false negative (FN) predictions. TP is the number of test segments correctly classified as labeled, and TN is the number of test segments correctly rejected for not belonging to the class. FP is the number of test segments classified into the wrong class, and FN is the number of test segments incorrectly rejected from the correct class. Since the data samples used were balanced, the accuracy was used as an evaluation measure for classification [28], which is calculated as follows:

(3) $$\mathrm{Accuracy}=\frac{\mathrm{TP}+\mathrm{TN}}{\mathrm{Total}\;\mathrm{trials}}$$

For verification analysis, we used false rejection rates (FRR), false acceptance rates (FAR), true acceptance rates (TAR), and true rejection rates (TRR) as defined in Equations (4)–(7). As we used the classification results to obtain the four verification metrics, it is not possible to compute the equal error rate (EER) of FA and FR, which is a popular metric in biometric authentication. Hence, half total error rate (HTER), as shown in Equation (8), was used as an equivalence to EER [65].

(4) $$\mathrm{FRR}=\frac{\mathrm{FN}}{\;\mathrm{Genuine}\;\mathrm{attempts}}=\frac{\mathrm{FN}}{\mathrm{FN}+\mathrm{TP}}$$

(5) $$\mathrm{FAR}=\frac{\mathrm{FP}}{\mathrm{Imposter}\;\mathrm{attempts}}=\frac{\mathrm{FP}}{\mathrm{FP}+\mathrm{TN}}$$

(6) $$\mathrm{TAR}=\frac{\mathrm{FN}}{\;\mathrm{Genuine}\;\mathrm{attempts}}=\frac{\mathrm{TP}}{\mathrm{TP}+\mathrm{FN}}$$

(7) $$\mathrm{TRR}=\frac{\mathrm{TN}}{\;\mathrm{Imposter}\;\mathrm{attmpts}}=\frac{\mathrm{TN}}{\mathrm{TN}+\mathrm{FP}}$$

(8) $$\mathrm{HTER}=\frac{\mathrm{FRR}+\mathrm{FAR}}{2}$$

## 5. Results and Discussion

In this section, we present the experimental results according to the experimental protocol discussed in Section 4.3. We also present comparisons and discussions about the results obtained.

### 5.1. Effect of Length and Segmentation of Signal on Classification Performance

The classification accuracy for different segments and lengths obtained from the PTB dataset (phase-1) is presented in this subsection. Table 4 shows the classification accuracy for blind segments of different lengths. The use of blind segmentation resulted in inconsistent samples that were difficult to learn from. Due to this problem, the trained classifier failed to obtain the desired recognition accuracy. According to Table 4, a smaller segment size leads to lower performance than a larger size. Using 2 s segmentation size results in segments containing approximately a single complete heartbeat. The 2 s segmentation size results in a 98.14% accuracy for GoogLeNet, approximately the same accuracy for the small CNN but a significantly lower accuracy for ResNet and much lower for EfficientNet and MobileNet. Among the tested lengths, 2 s produced the best result for blind segmentation.

Table 5 shows the classification accuracy for different, fiducial-based segmentations. It can be observed from the table that R-centered segments (especially segments of length 0.5 s) obtained a higher accuracy than P-P and R-R segments. This result indicates that a short segment around the R-peak could capture sufficient information for biometric recognition. Furthermore, the R-peak is the most apparent and recognizable point, which means that accurate identification of the R-peak ensures accurate and useful signal segmentation. Use of the P-P or R-R segment results in different segment lengths based on the distance between two consecutive P or R peaks, respectively. Hence, heartbeat resampling and alignment [66,67] are required to be used for biometric recognition. Using the P-peak for segmentation may not be convenient as the P-peak is difficult to identify, and the procedure used to remove noise and normalize the signal may affect the detection of the P-peak. It may produce inconsistent segments degrading the recognition performance.

**Figure 9:**
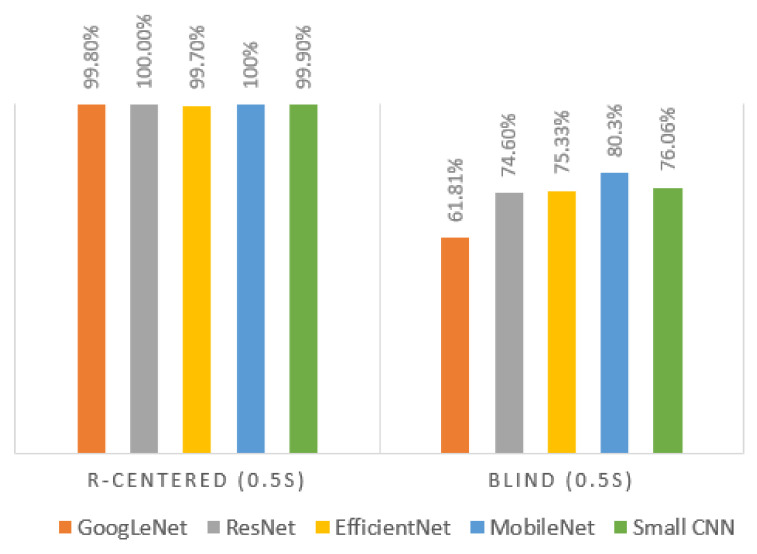
Classification performance for R-centered (0.5 s) segment vs. blind (0.5 s) segment.

While the 0.5 s blind segment gave the least accurate classification result, as shown in Table 4, the opposite result was obtained when the segmentation relied on the R-peak, as shown in Figure 9. It shows that the R-peak centered segment increased the accuracy by 30%, compared to blind segmentation, using all five different networks. The results presented in this section show that the combination of deep learning and the use of the right segment of the ECG signal can be effective for human recognition.

### 5.2. Biometric Recognition with Multisession Data

**Figure 10:**
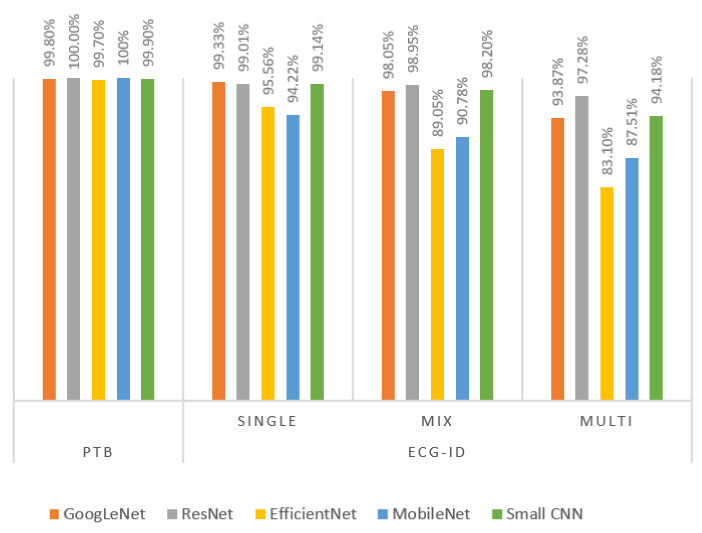
PTB and ECG-ID performance using single R-centered heartbeat.

The classification accuracy for using the 0.5 s segment captured around the R-peak obtained from the ECG-ID dataset (phase-2) is presented in this subsection. The experiments were conducted in different session scenarios as discussed in Section 4.3. The results for the session analysis are presented in Table 6 and Table 7. Table 6 shows the average accuracy for five different networks. Table 7 presents the accuracy of each fold of the 2-fold cross-validation in multisession scenarios and the average accuracy. It can be noted ResNet, GoogLeNet and small CNN yielded comparable results. On the other hand, the performance of EfficientNet and MobileNet was lower. Furthermore, the performance dropped significantly with the multisession records, and that can be due to the complexity of the networks. Figure 10 compares the accuracy for PTB and ECG-ID databases in different scenarios. It could be noted that the performance of multisession data is quite high, with accuracy exceeding 97% for the ResNet model.

### 5.3. Analysis of Identification and Verification Performance for Multisession Data

**Figure 11:**
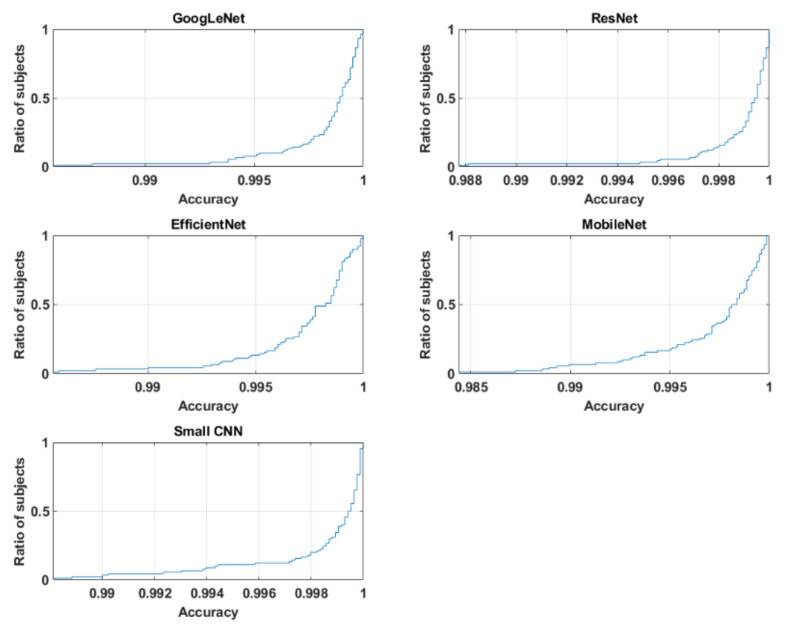
Cumulative distribution plot of accuracy for five networks.

**Figure 12:**
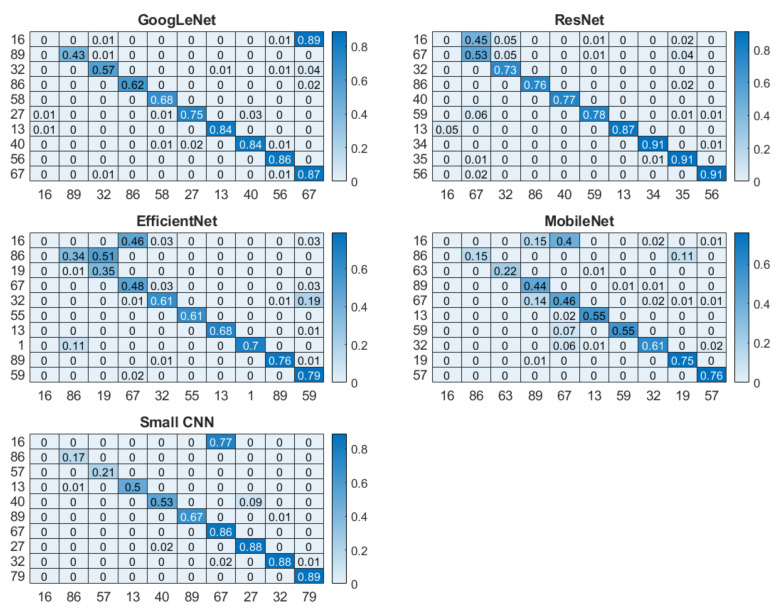
Subject-wise confusion matrix for five networks where the diagonal elements in darker color repsent the correct classification rate.

The overall recognition for multisession data was quite good for three networks, with 94.18%, 97.28% and 93.87% for small CNN, ResNet and GoogLeNet, respectively. However, EfficientNet and MobileNet did not perform that well; their accuracies were 83.10% and 87.51%, respectively. From the cumulative distribution plot of accuracy [64] as shown in Figure 11, it can be observed that most of the subjects had accuracy higher than 99%. In fact, for some of the subjects, it could be lower as shown in the subject-wise confusion matrix presented in Figure 12. As the number of subjects (90) is too high for meaningful visualization, the confusion matrix was sorted according to its diagonal value (true acceptance), and the values for only ten of the worst-performing subjects are shown. The Fisher Z-transformation was applied to calculate the population mean and standard deviation, yielding the mean accuracy and standard deviation for five networks as shown in Table 8.

**Figure 13:**
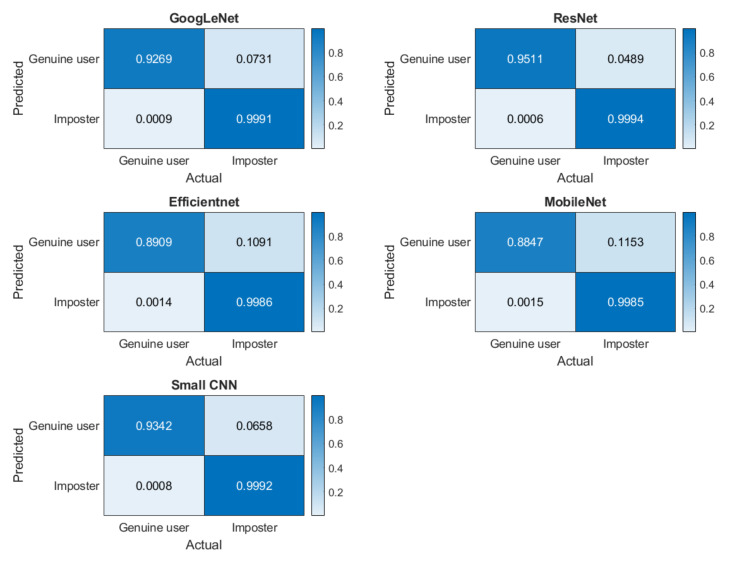
Confusion matrices for verification scenarios for five networks where the darker color elements represent correct classification rate.

For the verification scenario, the confusion matrixes for five networks are presented in Figure 13. The TRR, FRR, FAR, TAR, and HTER are shown in Table 9. We evaluated the confusion matrix statistically, using the McNemar test. The critical value at a 95% significance level is 3.8415 for all five networks. McNemar’s chi-square at alpha = 0.05 level is shown in Table 10.

Identification and verification analysis on multisession data reveals the strength of the short ECG segment on deep learning-based biometric recognition methods. Although most networks performed well, the proposed small network yielded the highest mean accuracy (Table 9), indicating that the quality of the segment is an important factor. The verification performances of all five networks are statistically significant (Table 10). Although for most of the subjects, the accuracy was high (Figure 13), only for few individuals the accuracy was low, as shown in the subject-wise confusion matrix in Figure 12. This could be due to the noisy signal captured in different sessions.

### 5.4. Comparison with State-of-the-Art Methods

We compared the results of our method with the state of the art for using both PTB and ECG-ID databases. Table 11 shows a comparison between the obtained results with state-of-the-art methods that tested their deep learning authentication systems, using the PTB database. Blind segmentation was found to be effective, using long signals with a small number of subjects, as in [24], where a segment length of 10 s was used to authenticate 52 subjects, resulting in 100% accuracy. However, for a larger number of individuals, as in our experiment, the fiducial-based, short segment gave a higher performance. We obtained 99.76, 100, 99.70, 100, and 99.83% accuracies through the use of 0.5 s segment lengths for GoogLeNet, ResNet, EfficientNet, MobileNet, and small CNN, respectively.

Table 12 shows a comparison between the obtained results with state-of-the-art methods that tested deep learning authentication systems, using the multisession ECG-ID database. From the table, it could be observed that the works that used fiducial-based heartbeat segmentation [49,52], along with the proposed model, performed better than works that used blind segmentation [37,50]. Although the model in [49] achieved 100% accuracy, it needs eight consecutive heartbeats. This accuracy decreased for using fewer beats, as when one heartbeat was used, the accuracy did not exceed 83.33%.

## 6. Conclusions

This paper investigated how the time–frequency domain representation of a short segment of an ECG signal could be effectively used for biometric recognition, using deep learning techniques to improve the acceptability of this modality. In most of the existing, deep learning-based recognition systems, a long segment of ECG signal is used to achieve high recognition accuracy. In contrast, we used a short segment of 0.5 s around the R-peak to obtain excellent recognition accuracy in multisession data, outperforming state-of-the-art methods. It can be concluded that time–frequency domain representation of a short segment of an ECG signal is important to increase the recognition capability, even for multisession data. In fact, less complex CNN models could be effective in biometric recognition, using a short segment of an ECG signal. The viability of time–frequency domain representation of the short segment can help to develop a robust, reliable, and acceptable authentication system useful for commercial and public applications.

To improve the reliability of the ECG signal as a biometric modality, further investigation is required to identify the performance of short invariant segments on larger multisession datasets. Moreover, the change in the signal due to different cardiac conditions is an important concern in the research community. For future work, we would like to investigate the recognition performance of the short segment under such circumstances. We would also like to develop a more sophisticated deep-learning machine that is robust to different changes of the signal over a long period.

## Figures and Tables

**Table 1:** State-of-the-art for ECG biometrics with database, learning model, segmentation type, and length, performance.

RefeRence	Dataset	Subjects	Segmentation		Classification	Performance
			Type	Length		
[32]	PTB	200	HB	0.66 s	CNN	Acc = 97.84%
[24]	PTB	52	Blind	10 s	CNN	Acc = 100%
[36]	CEBSDB	20	Blind	2 s	CNN	Acc = 99%
	STDB	28				Acc = 90.3%
	MI-BIH	47				Acc = 91.1%
	NSRDB	18				Acc = 95.1%
	AFDB	23				Acc = 93.9%
	WECG	22				Acc = 95.5%
	VFDB	22				Acc = 86.6%
	FANTASIA	20				Acc = 97.2%
[37]	ECG-ID	90	Blind	3 s	CNN	Acc = 96.63%
[38]	CYBHi	65	HB	0.8 s	CNN	EER = 13%
	UofTDB	46		3.5 s		
[39]	CEBSDB	20	HB	0.4 s	CNN	Acc = 93.1%
	NSRDB	18		1.5 s		Acc = 91.4%
	STDB	28		0.5 s		Acc = 92.7%
	AFDB	23		0.8 s		Acc = 89.7%
	FANTASIA	20		0.8 s		Acc = 99.9%
[27]	Privet	800	HB	3 s	CNN	EER = 2%
[41]	Mix (PTB+MIT-BIH)	175	Blind	15 s	SVM	Acc = 95.5%
[43]	Privet	460	HB	0.2 s, 1 s	LDA	Acc = 91.6%
[34]	PTB	10	HB (RR interval)	-	SVM	Acc = 97.45%
[47]	TEOAE	82	HB	11 s	SVM	EER = 6.9%
[45]	UofTDB	1019	HB	6 s	Euclidean Distance	EER = 5%
[44]	Privet	6	HB	0.65 s	SVM	Acc = 94.9%
[42]	PTB	50	Blind	10 s	Euclidean Distance	Acc = 100%
[46]	MIT-BIH	47	HB	0.5 s	Random Forest	Acc = 98%
	NSRDB	18		1.6 s		Acc = 99%
	ECG-ID	90		2 s		Acc = 91%
[50]	FANTASIA	20	Blind	6 s	CNN	Acc = 99.98%
	ECG-ID (Multi)	90		4 s		Acc = 73%
	ECG-ID (Single)					Acc = 94.23%
[40]	PTB	52	HB (RR interval)	-	CNN	Acc = 98.45%
	MIT-BIH	18				Acc = 99.2%
[51]	ECG-ID	90	HB	0.6 s	GRU	Acc =98.6%
	MIT-BIH	47		0.5 s		Acc = 98.4%
[52]	PTB	52	HB	1.2 s	CNN	Acc =100%
	ECG-ID	90				Acc = 98.24%
	MIT-BIH	47				Acc = 95.99%
[49]	ECG-ID	90	HB	0.5 s × 8	CNN	Acc = 100%
	MIT-BIH	47		0.5 s × 6		
[32]	ECG-ID	89	HB	0.5 s × 9	LSTMGRU	Acc = 100%
	MIT-BIH	47		0.6 s × 9		ERR = 3.5%
[33]	Privet	140	HB (RR interval)	-	SVM	Acc = 93.15%
	PTB	50				

**Table 2:** Proposed small CNN architecture.

Layer Number	Type	Input Size	Number of Filters	Size of Filters	Stride	Padding
1	Image Input	224 × 224 × 3	-	-	-	-
2	Convolution	224 × 224 × 3	32	7 × 7	1	3
3	Max Pooling	224 × 224 × 32	-	2 × 2	2	1
4	ReLU	113 ×1 13 × 32	-	-	-	-
5	Batch Normalization	113 × 113 × 32	-	-	-	-
6	Convolution	113 × 113 × 32	32	3 × 3	1	1
7	Convolution	113 × 113 × 32	32	3 × 3	1	1
8	Batch Normalization	113 × 113 × 32	-	-	-	-
9	Addition	113 × 113 × 32	-	-	-	-
10	ReLU	113 × 113 × 32	-	-	-	-
11	Convolution	113 × 113 × 32	64	3 × 3	2	1
12	Convolution	57 × 57 × 64	64	3 × 3	2	1
13	Batch Normalization	29 × 29 × 64	-	-	-	-
14	Convolution	113 × 113 × 32	64	1 × 1	2	0
15	Max Pooling	57 × 57 × 64	-	2 × 2	2	1
16	Addition	57 × 57 × 64	-	-	-	-
17	Batch Normalization	57 × 57 × 64	-	-	-	-
18	Convolution	57 × 57 × 64	128	3 × 3	2	1
19	Convolution	15 × 15 × 128	128	3 × 3	2	1
20	Batch Normalization	8 × 8 × 28	-	-	-	-
21	Convolution	57 × 57 × 64	128	1 × 1	2	0
22	Max Pooling	15 × 15 × 128	-	2 × 2	2	1
23	Addition	8 × 8 × 28	-	-	-	-
24	Global Max Pooling	8 × 8 × 28				
25	Fully Connected	1 × 1	Number of Class	-	-	0
26	Softmax	1 × 1	Softmax	-	-	-
27	Classification Output	-	-	-	-	-

**Table 3:** Number of layers, learnables (M = Million, K = Thousand) and average training for each model.

Network	Number of Layers	Learnable Parameters	Average Training Time
GoogLeNet	144	5.9 M	132 min
ResNet	71	4.8 M	48 min
EfficientNet	290	4.1 M	112 min
MobileNet	154	2.4 M	53 min
Small CNN	27	324 K	37 min

**Table 4:** Blind segmentation performance with different lengths of a signal.

Length of Signal (second)	Accuracy (%)				
	GoogLeNet	ResNet	EfficientNet	MobileNet	Small CNN
0.5	61.81	74.6	75.33	80.3	76.06
1	97	87.2	57.30	61.3	97.84
1.5	98.10	92.7	62.10	63.7	98.82
2	98.14	93.2	63.05	64	98.90
2.5	97.61	93.9	62.12	63.54	96.0
3	95.77	94.9	58.03	60.86	93.0

**Table 5:** Classification accuracy for single heartbeat using different types of segmentation.

Segmentation	Length of Signal (seconds)	Accuracy (%)				
		GoogLeNet	ResNet	EfficientNet	MobileNet	Small CNN
R-centered	0.5 s	99.90	100	99.70	100	99.90
	0.75 s	99.34	99.96	99.32	99.92	99.61
	1 s	99.25	99.65	99.01	99.70	99.52
P-P	1 HB	98.50	97.39	92.1	93.9	97.86
R-R	1 HB	98.83	99.30	96.98	97.5	98.0

**Table 6:** ECG-ID session analysis performance.

Session Scenario	Accuracy (%)				
	GoogLeNet	ResNet	EfficientNet	MobileNet	Small CNN
Single	99.33	99.01	95.56	94.22	99.14
Mix	98.05	98.95	89.05	90.78	98.20
Multi	93.87	97.28	83.10	87.51	94.18

**Table 7:** Detailed ECG-ID multisession scenario performance.

Sessions		Accuracy (%)				
Testing	Training	GoogLeNet	ResNet	EfficientNet	MobileNet	Small CNN
S1	S2	95.06	96.78	89.09	88.47	95.54
S2	S1	92.68	97.78	77.12	86.56	92.81
Average		93.87	97.28	83.10	87.51	94.18

**Table 8:** The mean accuracy and standard deviation for five networks.

Network	Mean Accuracy	Standard Deviation
Small CNN	0.9511	0.1231
ResNet	0.9342	0.1720
GoogLeNet	0.9269	0.1359
EfficientNet	0.8909	0.1596
MobileNet	0.8847	0.1817

**Table 9:** The TRR, FRR, FAR, TAR, and HTER for five networks.

Network	TRR	FRR	FAR	TAR	HTER
Small CNN	0.9992	0.0008	0.0658	0.9342	0.033
ResNet	0.9994	0.0006	0.0489	0.9511	0.025
GoogLeNet	0.9991	0.0009	0.0731	0.9296	0.037
EfficientNet	0.9986	0.0014	0.1019	0.8909	0.052
MobileNet	0.9985	0.0015	0.1153	0.8847	0.058

**Table 10:** McNemar chi-square with alpha = 0.05 level.

Network	Yates Correction	*p*
Small CNN	0.000845	0.976815
ResNet	0.001136	0.973108
GoogLeNet	0.000760	0.978008
EfficientNet	0.000509	0.981998
MobileNet	0.000482	0.982490

**Table 11:** Comparison of results with state-of-the-art methods that used CNN and PTB dataset.

Refrance	Number of Subjects	Length of Signal (second)	Segmentation Method	Accuracy (%)
[32]	200	0.66	HB	97.84
[24]	52	10	Blind	100
[52]	52	1.2 (2 HBs)	HB	100
GoogLeNet	100	0.5	HB	99.76
ResNet				100
EfficientNet				99.70
MobileNet				100
Small CNN				99.83

**Table 12:** Comparison of results with state-of-the-art methods that use CNN and ECG-ID.

Refrance	Number of Subjects	Session	Length of Signal (seconds)	Segmentation Method	Accuracy (%)
[52]	90	Single	1.2 (2 HBs)	HB	98.24
[37]	50	Multi	3	Blind	96.63
[49]	90	Multi	0.5 × 1	HB	83.33
			0.5 × 8		100
[50]	90	Mix	4	Blind	94.23
		Multi			73.54
GoogLeNet	90	Single	0.5 (1 HB)	HB	99.33
ResNet					99.01
EfficientNet					95.56
MobileNet					94.22
Small CNN					99.14
GoogLeNet	90	Mix	0.5 (1 HB)	HB	98.05
ResNet					98.95
EfficientNet					89.05
MobileNet					90.78
Small CNN					98.2
GoogLeNet	90	Multi	0.5 (1 HB)	HB	93.87
ResNet					97.28
EfficientNet					83.10
MobileNet					87.51
Small CNN					94.18

## Data Availability

Databases used in this study are available online.
